# ssAAVs containing cassettes encoding SaCas9 and guides targeting hepatitis B virus inactivate replication of the virus in cultured cells

**DOI:** 10.1038/s41598-017-07642-6

**Published:** 2017-08-07

**Authors:** Tristan Scott, Buhle Moyo, Samantha Nicholson, Mohube Betty Maepa, Koichi Watashi, Abdullah Ely, Marc S. Weinberg, Patrick Arbuthnot

**Affiliations:** 1 Wits/SAMRC Antiviral Gene Therapy Research Unit, Health Sciences Faculty, University of the Witwatersrand, Johannesburg, South Africa; 2 HIV Pathogenesis Research Unit, Health Sciences Faculty, University of the Witwatersrand, Johannesburg, South Africa; 3 National Institute of Infectious Diseases, Department of Virology II, Tokyo, Japan

## Abstract

Management of infection with hepatitis B virus (HBV) remains a global health problem. Persistence of stable covalently closed circular DNA (cccDNA) during HBV replication is responsible for modest curative efficacy of currently licensed drugs. Novel gene editing technologies, such as those based on CRISPR/Cas9, provide the means for permanently disabling cccDNA. However, efficient delivery of antiviral sequences to infected hepatocytes is challenging. A limiting factor is the large size of sequences encoding Cas9 from *Streptococcus pyogenes*, and resultant incompatibility with the popular single stranded adeno-associated viral vectors (ssAAVs). We thus explored the utility of ssAAVs for delivery of engineered CRISPR/Cas9 of *Staphylococcus aureus* (Sa), which is encoded by shorter DNA sequences. Short guide RNAs (sgRNAs) were designed with cognates in the *S* open reading frame of HBV and incorporated into AAVs that also encoded SaCas9. Intended targeted mutation of HBV DNA was observed after transduction of cells with the all-in-one vectors. Efficacy against HBV-infected hNTCP-HepG2 cells indicated that inactivation of cccDNA was successful. Analysis of likely off-target mutagenesis revealed no unintended sequence changes. Use of ssAAVs to deliver all components required to disable cccDNA by SaCas9 is novel and the technology has curative potential for HBV infection.

## Introduction

Chronic infection with hepatitis B virus (HBV) is responsible for a significant global disease burden^1^. There are currently about 240 million HBV chronic carriers in the world, and these individuals have an increased risk for life-threatening complications of cirrhosis and hepatocellular carcinoma. HBV gains access to hepatocytes through interaction with the human Na-taurocholate cotransporting polypeptide (hNTCP) receptor^2, 3^. Upon entry of virions into hepatocytes, relaxed circular DNA (rcDNA) is transported to the nucleus where it is released and repaired to form covalently closed circular DNA (cccDNA)^4^. Viral protein-coding mRNAs and pre-genomic RNA (pgRNA) are produced from this replication intermediate. pgRNA is the template for reverse transcription and formation of rcDNA then cccDNA. Currently licensed therapies, which include interferon-alpha derivatives, as well as nucleoside and nucleotide analogs, suppress viral replication but rarely eradicate the cccDNA reservoir. Therefore, focus has recently been placed on advancing potentially curative treatment approaches that are capable of eliminating HBV by disabling cccDNA permanently.

Several studies have explored the potential of using gene editing technologies to target cccDNA^5^. Zinc-finger nucleases (ZFNs)^6^, transcription activator-like effector nucleases (TALENs)^7^, and clustered regularly interspaced palindromic repeats (CRISPR) with CRISPR-associated protein (Cas9)^8^ have all been used in an attempt to inactivate cccDNA permanently. The rationale underlying use of gene editing to inactivate cccDNA is based on activation of the error-prone non-homologous end joining (NHEJ) repair pathway. Repeated cleavage of cccDNA by the gene editors followed by NHEJ eventually leads to targeted formation of insertions and deletions (indels) at the cognate site of the gene editors. The simplicity of employing RNA-guided CRISPR/Cas9 has allowed for its use in numerous applications, which include targeted disruption of cccDNA. There is growing evidence indicating that cccDNA may be mutated and in some cases eradicated using CRISPR/Cas9^9–11^. Although application of CRISPR/Cas9 to treating chronic HBV infection has shown promise, efficient and safe delivery of the RNA-guided nucleases (RGNs) to HBV-infected hepatocytes remains a significant challenge.

Recombinant single stranded adeno-associated viruses (ssAAVs) are attractive vectors for gene therapy of liver diseases^12^. Typically there is a low immune response to AAVs following their administration *in vivo*. Pathology in humans caused by the parent parvoviruses has not been reported and their AAV derivatives have little toxicity. The molecular biology underlying generation of AAVs is now well understood. It is possible to alter capsid sequences of the vectors to confer desirable biological properties, such as tropism for particular tissues. A drawback of ssAAVs is that the size of the transgene that may be accommodated is limited to approximately 4.5 kb, and this is further reduced in self-complementary AAVs (scAAVs). This is particularly important for development of potentially therapeutic vectors that deliver sequences comprising gene editors derived from engineered CRISPR/Cas9. Cas9 from *Streptococcus pyogenes* (SpCas9) has been the most widely used and has been shown to inactivate HBV sequences effectively^13^. However, the open reading frame (ORF) of SpCas9 comprises approximately 4.1 kb. Together with regulatory sequences and a short guide RNA (sgRNA) cassette, elements comprising the Sp system are too large to be accommodated by one ssAAV. Various approaches have been employed to address this limitation of ssAAVs. These include exploiting the bilobed structure of SpCas9 and use of two vectors that together express a ‘scarless’ SpCas9^14^. Generic methods of increasing ssAAV capacity, such as by using homologous recombination between overlapping sequences of different ssAAVs (reviewed in ref. 15), may also be useful. Although the approaches have been employed successfully, the requirement for more than one ssAAV complicates the methodology.

The recently described orthologous Cas9 that is derived from *Staphylococcus aureus* (SaCas9) is approximately 1 kb smaller than SpCas9^16^. Sequences encoding SaCas9 as well as regulatory elements and the sgRNA cassette have been packaged into an ssAAV and used to edit endogenous genes in the liver. This ‘all-in-one’ system, which overcomes the need for using more than one AAV, has potential for application to therapeutic gene editing and may be useful for inactivation of genes of HBV. The approach has been used successfully to mutate *CCR5*, a host dependency factor that is required for HIV-1 infection by CCR5-tropic viruses^17^. To advance use of CRISPR/Cas9-based technology for treatment of HBV infection, we incorporated cassettes encoding SaCas9 and sgRNAs into ssAAVs. Targeting the HBV *S* open reading frame (ORF) resulted in efficient inactivation of HBV replication and mutation of cccDNA in cultured cells.

## Results

### Identification of an effective anti-HBV *SaCas*9 sgRNA

**Figure 1:**
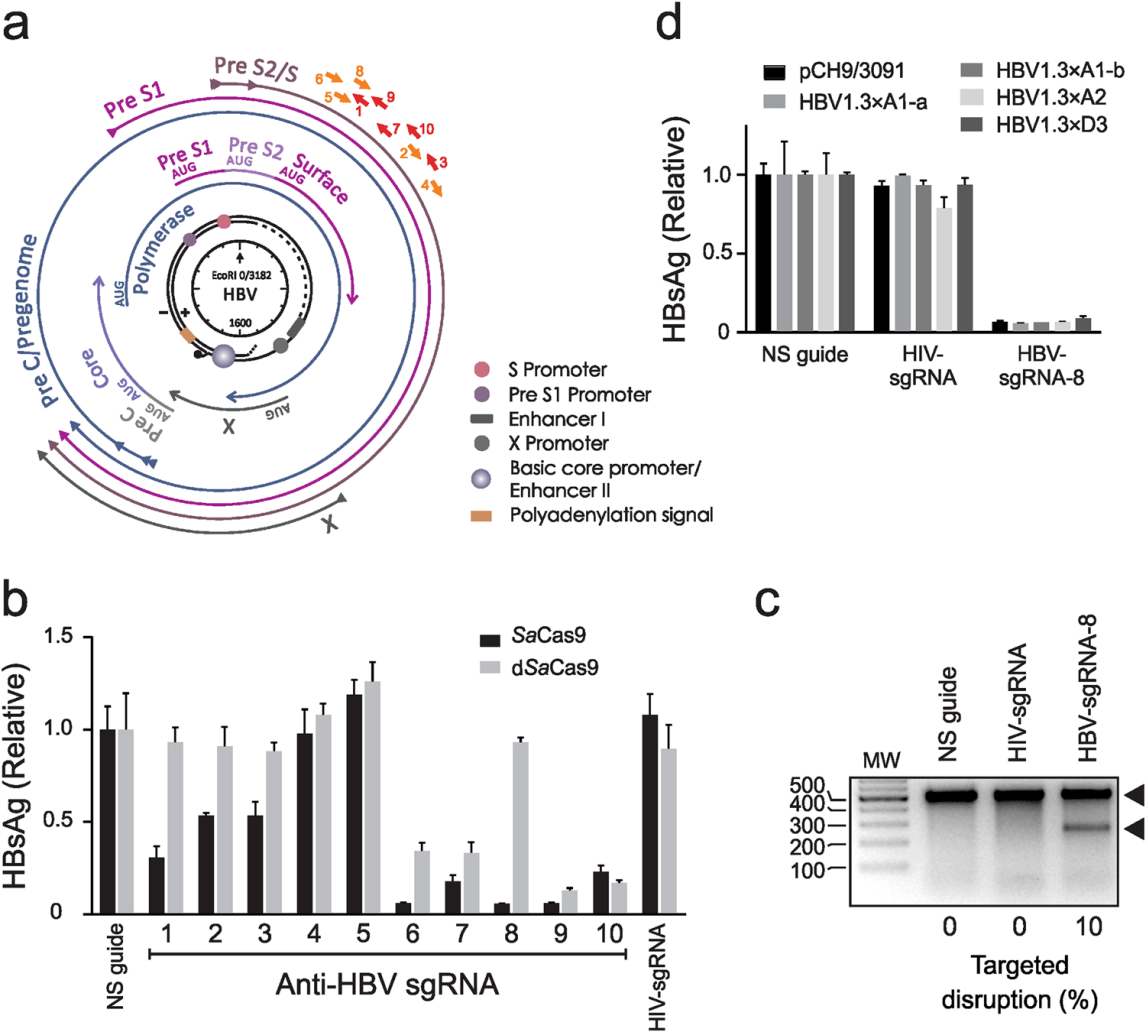
Screening of panel of anti-HBV SaCas9-sgRNAs. (**a**) Schematic illustration of the HBV genome with indicated open reading frames (ORFs). The viral rcDNA, with regulatory *cis* elements, is indicated at the center and coordinates are given relative to the single *Eco*RI site. The four HBV ORFs are shown as arrows immediately surrounding the genome and the four major viral transcripts are indicated as outermost arrows. The sgRNA target sites are depicted as arrows encompassing most of the sequence of the *surface* (*S*) region of the virus. (**b**) Huh7 cells were co-transfected with plasmids encoding i) a replication competent molecular clone of HBV (pCH-9/3091), ii) each of a panel of cassettes expressing *S*-targeting single guide RNAs (sgRNAs) and iii) either SaCas9 or catalytically dead SaCas9 (dSaCas9) expressed from a CMV promoter/enhancer element. The concentrations of HBV S antigen were measured using an ELISA and data are presented relative to the values for the mean for a non-specific sgRNA sequence. A sgRNA targeted to HIV (HIV-sgRNA) was included as an additional negative control. Data are represented as the means and the error bars indicate standard deviation. (**c**) HEK293T cells were transfected with SaCas9 and sgRNA-8 with pCH-9/3091. A T7E1 assay was performed on extrachromosomal cellular DNA. Arrows indicate the larger intact PCR product (~550 bp) and smaller digested fragment (~280 bp). The measured target disruption is indicated below as a percentage. (**d**) Huh7 cells were transfected with SaCas9 and sgRNA-8 targeting a greater than genome length molecular clone (1.3 × ) for HBV sub-genotypes A1, A2 and D3. Data are presented as in b.

A panel of sgRNAs was designed to target the small *surface* (*S*) ORF of HBV that overlaps the *polymerase* (*P*) gene of HBV (Fig. 1a). The S protein is a viral envelope glycoprotein that is essential for viral entry and infectious virion formation. P carries out a vital role in reverse transcription of pgRNA and replication of the viral DNA. Target sites were selected on the basis of conserved status amongst HBV isolates and their being distinct from human genomic sequences. The 20 bp cognates of the sgRNAs were each common to all HBV genotypes and analysis using the Off-Spotter tool^18^ showed that each guide had a minimum of two mismatches with possible off-target sites of the human genome. Ten target sites were selected and guide cassettes were constructed to express sgRNAs as a single transcript comprising the SaCas9 tracrRNA and sequence of 20 nucleotides complementary to the HBV target sequence. To evaluate anti-HBV efficacy, DNA encoding SaCas9, sgRNAs and an HBV replication-competent plasmid were initially transfected into Huh7 cells^19^. Eight of the ten sgRNAs reduced concentrations of HBV S antigen (HBsAg) in the culture supernatants by 50–95% when compared to controls, which were a non-specific sgRNA or an sgRNA targeted to HIV (Fig. 1b). Since nuclease activity is required for potent and subsequent mutation of HBV cccDNA, catalytically dead SaCas9 (dSaCas9) was included as an additional control. Transfection of plasmids encoding each of sgRNA-6, -7, -9 and -10 reduced HBsAg in the culture supernatants when co-transfected with the dSaCas9 sequence. These data suggest that some of the repressive activity resulted from transcriptional interference, rather than from being caused by DNA cleavage and formation of indels following error-prone NHEJ. Conversely sgRNA-8 was inactive with dSaCas9 but effectively reduced HBsAg concentrations in the presence of nuclease-competent SaCas9. This indicated that the inhibitory effect of sgRNA-8 on release of the HBV replication marker from cultured cells resulted from cleavage-mediated mutagenesis. An assay using T7 endonuclease 1 (T7E1) to analyze re-annealed amplicons covering the sgRNA-8 target confirmed indel formation by SaCas9 with this guide (Fig. 1c). sgRNA-8 was thus identified as the lead and was selected for further investigation.

To evaluate whether sgRNA-8 could target multiple sub-genotypes of HBV, DNA encoding the cassette was co-transfected with replication-competent clones of sub-genotypes A1-a, A1-b, A2 and D3^20^ (Fig. 1d and Supp. Fig. 1). Suppression of S antigen was efficient and dependent on presence of catalytically active saCas9. The efficacy was similar to that achieved when sequences encoding the RGN were co-transfected with pCH-9/3091, a genotype D-derived sequence (Fig. 1b and d). The target of sgRNA-8 is also conserved in other HBV isolates (Supp. Table 1) and indicates that the gene editor should be active against most variants of the virus.

### Evaluating inhibition of HBV replication in cultured HepG2.2.15 cells using an ssAAV to deliver cassettes encoding SaCas9 and sgRNA-8

**Figure 2:**
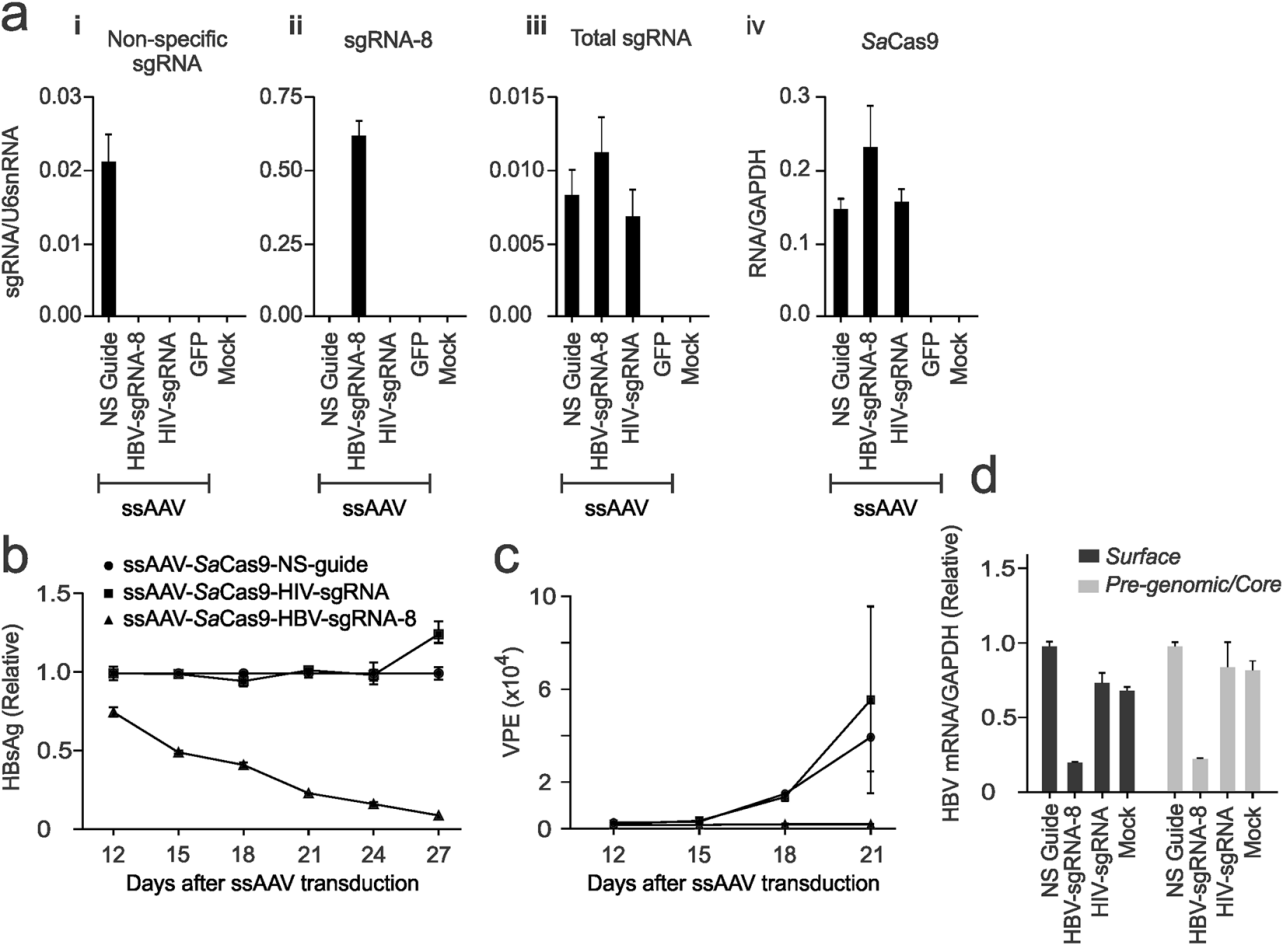
Inhibition of markers of HBV replication following delivery of SaCas9-sgRNA-8 with ssAAVs. (**a**) HepG2.2.15 cells were transduced with ssAAVs that encoded HBV-targeting sgRNA-8, a non-specific guide, HIV-targeting sgRNA or a GFP reporter. RNA was extracted 21 days after transduction and subjected to qRT-PCR. Primers were complementary to (i) sgRNA-8, (ii) the non-specific control sgRNA, (iii) all sgRNAs or (iv) SaCas9 sequences. (**b**) Concentrations of HBsAg were measured in the culture supernatants of cells that had been transduced with ssAAVs encoding SaCas9 and sgRNA-8, sgRNA targeting HIV or a non-specific sequence. Analysis was carried out over a time course of 27 days after transduction. (**c**) Assay of viral particle equivalents (VPEs) in culture supernatants of HepG2.2.15 cells after transduction with HBV-targeting and control ssAAVs. Cells were washed and measurements were based on qPCR carried out on extracts of culture supernatants taken from days 12 to 21 after transduction. (**d**) HBV RNA was measured in transduced cells at day 21 after transduction with the indicated ssAAVs. qRT-PCR was carried out with primers specific for *surface* (*S*) or *pregenomic* HBV RNA sequences.

Both cassettes of SaCas9 and sgRNA-8 were incorporated into an ssAAV to assess functionality of the all-in-one vector. The sequences comprising the RGN were packaged into an AAV2 vector, termed ssAAV-SaCas9-sgRNA-8, and used to transduce HepG2.2.15 cells^21^, which replicates HBV from a stable integrant. With these all-in-one vectors, the sgRNAs were each transcribed from a U6 Pol III promoter. The SaCas9 sequence was expressed from a separate cassette regulated by a CMV Pol II enhancer and promoter element. Similar concentrations of SaCas9 mRNA and different sgRNAs were verified using qRT-PCR (Fig. 2a), which indicates efficient expression of each component of the RGNs when produced from various combinations in individual ssAAV2s. After treatment of HepG2.2.15 cells with ssAAV-SaCas9-sgRNA-8, there was a rapid reduction in the concentration of the HBsAg in the culture supernatant. Baseline levels were reached over the 27 day period of the investigation (Fig. 2b). Conversely the control sgRNAs did not cause inhibition of secretion of the marker of HBV replication. Similarly, secretion of HBV viral particle equivalents (VPEs), as measured using qPCR to quantitate viral DNA in the culture supernatants, was undetectable in the ssAAV-SaCas9-sgRNA-8-treated cells (Fig. 2c). Analysis of HBV RNA revealed a reduction of approximately 80% of viral *S* and pgRNA transcripts (Fig. 2d).

**Figure 3:**
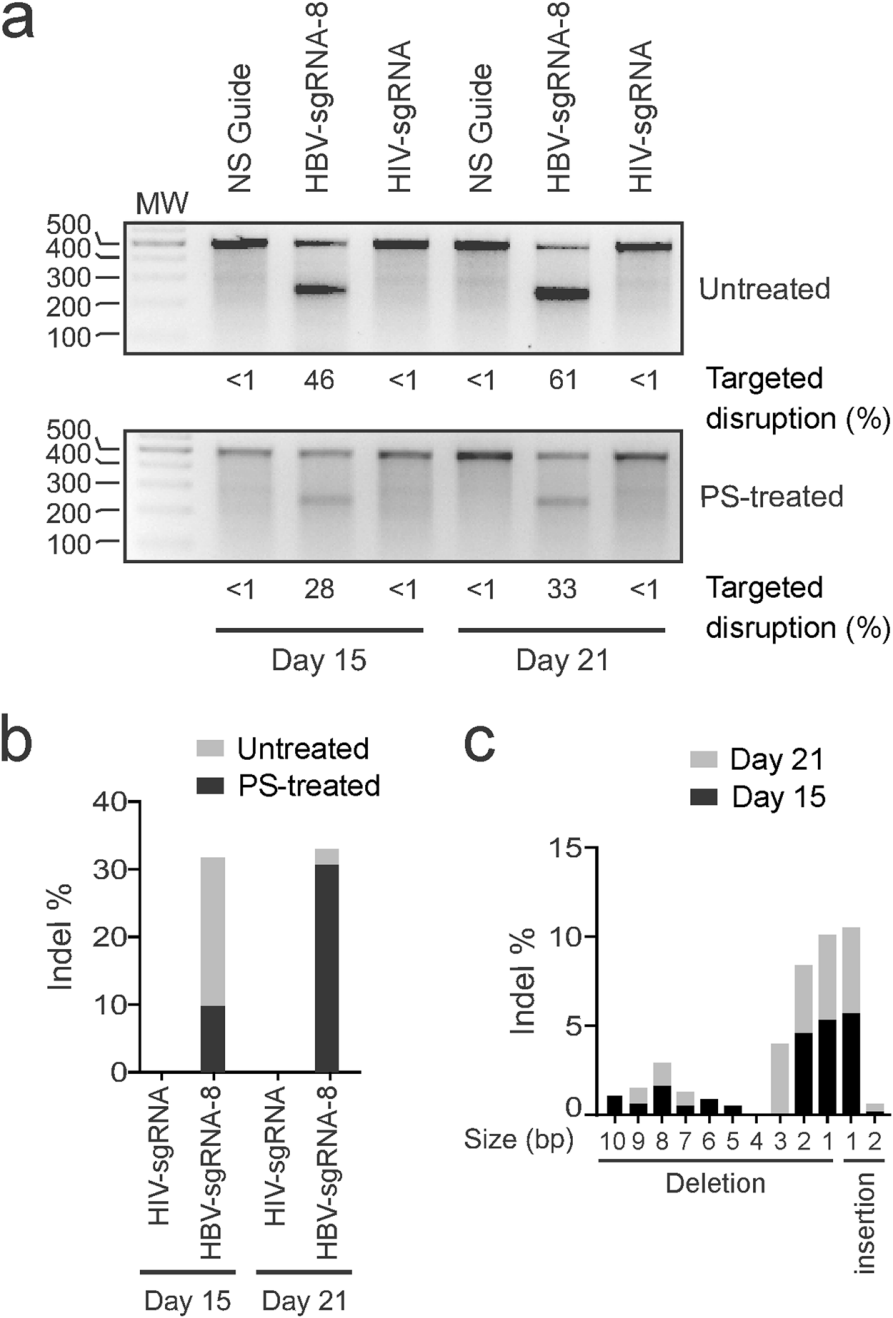
Targeted mutation of HBV DNA by AAV2-SaCas9-sgRNA-8. (**a**) Total DNA extracted from ssAAV-transduced cells with and without plasmid-safe (PS) treatment was subjected to T7E1 analysis to detect insertions and deletions (indels) in viral DNA. The percentages of HBV DNA sequences containing targeted mutations at 15 and 21 days after transduction are indicated for sgRNA-8 and the two control guides. (**b**) Drop-off ddPCR™ assay to detect indels in ssAAV-transduced cells. Analysis was performed 15 and 21 days after transduction with AAV2-SaCas9-sgRNA-8 or the HIV-targeting control vector. (**c**) Tracking of indels by decomposition (TIDE) was carried out on raw Sanger sequencing chromatograms that were generated using amplicons of viral sequences as the template. Sizes of the predicted indels, together with the proportions of the total that they represent, are presented. Analysis was carried out on samples extracted from cells that had been transduced at 15 and 21 days after transduction with ssAAVs.

Formation of indels in the target viral DNA was evaluated using a T7E1 assay and drop-off droplet digital PCR (ddPCR^TM^)^22^. The T7E1 assay revealed that 46% of targets had indels by day 15 after treatment with ssAAV-SaCas9-sgRNA-8, and the proportion of mutated sequences increased to 61% by day 21 (Fig. 3a). The drop-off ddPCR^TM^ analysis demonstrated indel formation in approximately 30% of the targets by day 21 (Fig. 3b). The difference between results obtained for the T7E1 and ddPCR^TM^ assays may be a result of a more stringent but less sensitive nature of the ddPCR^TM^ assay. Tracking of indels by decomposition (TIDE), which uses a decompression algorithm to decode automated sequencing, was employed to analyze the types of mutations at the target site^23^. Results showed a strong bias towards single or double base pair deletions and single insertions (Fig. 3c). These mutations continued to increase from day 15 to day 21. Importantly, such sequence changes would cause frame shift mutations and major disruption to expression of HBV genes. To evaluate effects on cccDNA, extracts from HepG2.2.15 cells were treated with plasmid-safe DNase to eliminate cellular DNA, rcDNA and linear DNA containing viral sequences^7, 24^. As previously described, control murine samples lacking cccDNA, but abundant in other forms of HBV DNA, were used to verify elimination of non-cccDNA. Indels were detected in these preparations of cccDNA, although at lower levels than in the DNA extracts that were not treated with plasmid-safe. The T7E1 assay revealed mutation of 28% of targets of viral sequences at day 15 and indel formation was 33% by day 21 (Fig. 3a). These results were corroborated by using the drop-off ddPCR^TM^ analysis. Mutations may be caused by action of CRISPR/SaCas9 on viral integrants, and transmission of the changed sequences to pgRNA, rcDNA then cccDNA. Alternatively mutations detected in cccDNA may result from direct action of sgRNA-8 and saCas9 on this viral replication intermediate. Evidence of mutation of cccDNA prompted investigation of efficacy of the ssAAV-SaCas9-sgRNA-8 in cultured liver-derived cells that are infectable by HBV.

### Eradication of cccDNA from HBV-infected hNTCP-HepG2

**Figure 4:**
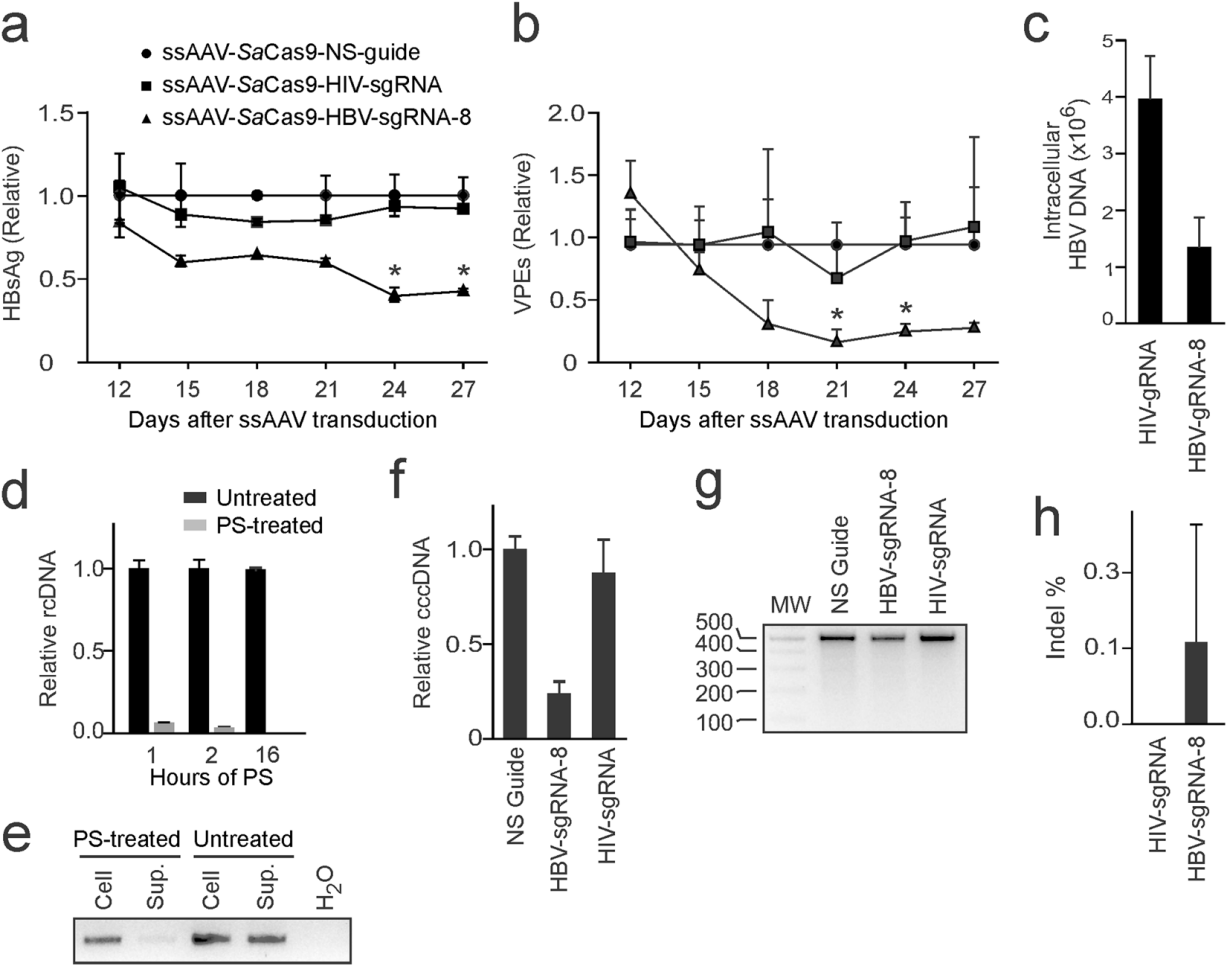
Evaluation of targeted mutation of cccDNA in HBV-infected hNTCP-HepG2 cells. Infection with HBV was carried out 24 hours prior to transduction of hNTCP-HepG2 cells with ssAAVs. Concentrations of (**a**) HBsAg and (**b**) viral particle equivalents (VPEs) in the culture supernatant were determined as described in Fig. 2b and c, respectively. Data are represented as the means and the error bars indicate standard deviation. Statistical differences are indicated by an asterisk (*p < 0.05). (**c**) Intracellular HBV DNA was measured on day 27 after transduction of cells with the indicated ssAAVs. (**d**) DNA from culture supernatants of hNTCP-HepG2 cells was extracted with or without treating with plasmid-safe DNase for the indicated times and subjected to analysis using qPCR with primers specific for HBV DNA. (**e**) Representative agarose gel electrophoresis to detect HBV DNA from cell extracts and supernatants with or without treatment with plasmid-safe DNase. (**f**) Total DNA was extracted from HBV-infected hNTCP-HepG2 cells at day 27 after transduction with ssAAVs. cccDNA was quantified using qPCR and values are presented relative to the mean for the control non-specific guide RNA. Detection of indels in cccDNA using (**g**) T7E1 or (**h**) drop-off ddPCR^TM^ assays. Comparisons were made using a two-tailed Student’s t-test, and significant statistical differences to the control samples treated with the non-specific guide are indicated (*p < 0.05).

To evaluate an effect of the combination of SaCas9 and sgRNA-8 on cccDNA more precisely, the hNTCP-HepG2 cell line was used as a model. Stable expression of NTCP in the HepG2-derived cells enables them to be infected with HBV^2^. The importance of using the model to evaluate efficacy of ssAAV-SaCas9-sgRNA-8 is that HBV gene expression in the infected cells is derived from cccDNA^2^, and not an integrated sequence as is the case with the HepG2.2.15 line. Inhibition of HBV replication following transduction of hNTCP-HepG2 cells by the RGN encoding ssAAV may thus be directly ascribed to an effect on cccDNA. hNTCP-HepG2 cells were thus infected with HBV prior to treatment with the ssAAV-SaCas9-sgRNA vectors. By day 27, concentrations of HBsAg steadily decreased to 55% of that measured at the start (Fig. 4a and Supp. Fig. 2), secreted VPEs were reduced by approximately 60% (Fig. 4b) and there was an approximate fourfold decline in total intracellular HBV DNA (Fig. 4c).

To analyze mutagenic effects on cccDNA from HBV-infected hNTCP-HepG2 cells, DNA was extracted from these cells after transduction. Extended digestion with plasmid-safe nuclease eliminated the detectable rcDNA and cellular genomic DNA, indicating that the remaining HBV DNA comprised cccDNA (Fig. 4d and e). Quantification of cccDNA at day 27 showed an approximately 80% reduction in this DNA species after treatment of cells with ssAAV-SaCas9-sgRNA-8 (Fig. 4f). A decrease in intracellular cccDNA concentrations has previously been reported after treatment of HBV replicating cells with virus-targeting SpCas9 and guide sequences^25, 26^. Interestingly, analysis using T7E1 and drop-off ddPCR^TM^ revealed no or little indel formation (Fig. 4g and h). Collectively the data suggest that the Sa-derived RGNs preferentially cause degradation of cccDNA. Substrates for the T7E1 and drop-off ddPCR^TM^ assays may comprise the remaining population of wild type sequences, which would account for the low indel detection.

### Analysis of off-target effects of SaCas9-sgRNA-8

**Figure 5:**
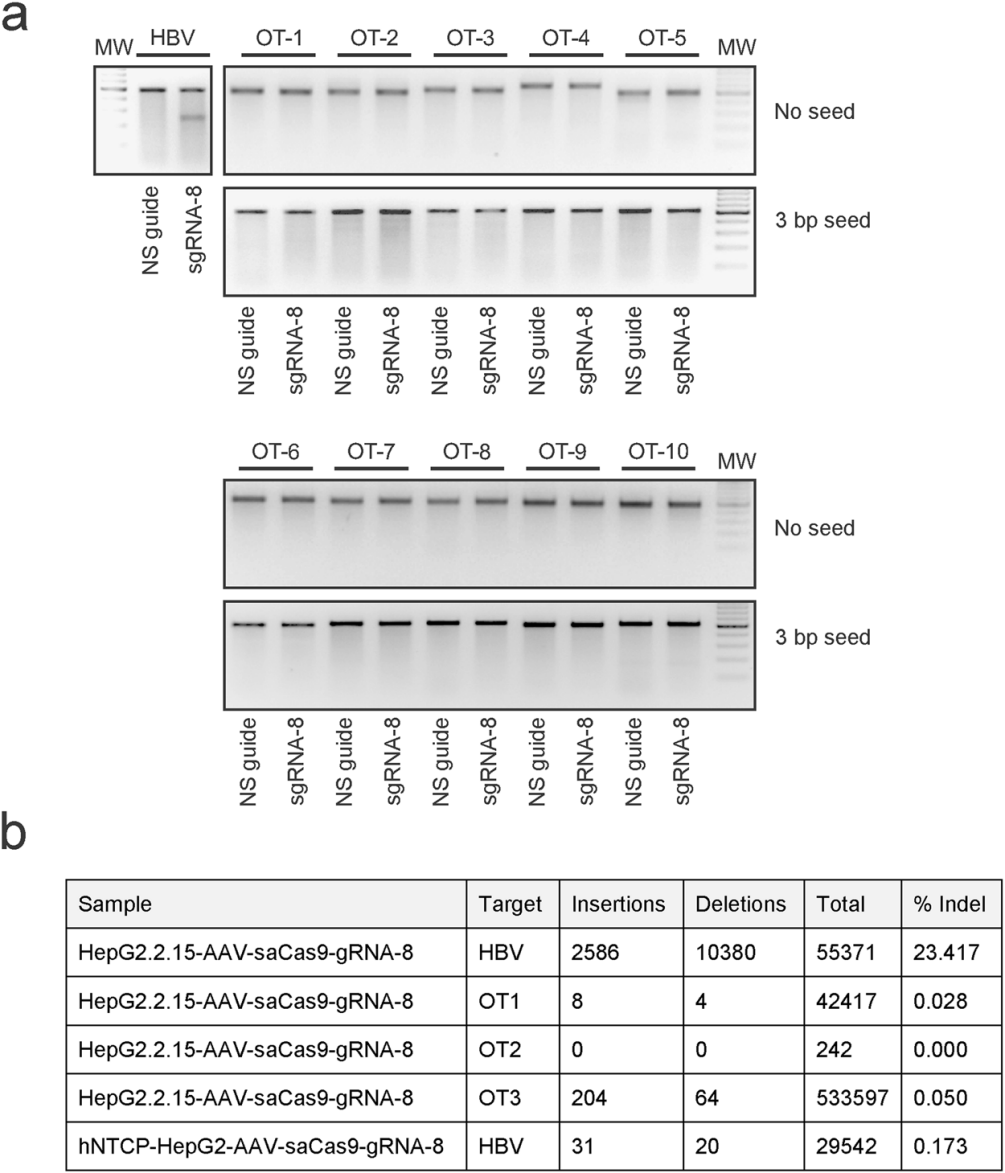
Evaluation of SaCas9-sgRNA8 off-target activity. (**a**) The top ten off-target sites were identified using the Off-spotter online tool with either a no seed prediction or with a fixed 3 bp seed panel. HEK293 cells were transfected with the SaCas9 vector and sgRNA-8 or non-specific guide control. Genomic DNA was extracted 48 hours after transfection, and a T7E1 assay was performed on the off-target sites. pCH-9/3091 was included to verify an HBV on-target signal. (**b**) Next generation sequencing analysis to detect insertions and deletions at on-target and off-target sites. The number of reads with insertions and deletions are indicated and the percentage of indels (% Indel) is calculated from the total reads.

Mutation at untargeted sites within the host genome may have serious consequences for potentially therapeutic RGNs. Analysis of unintended sequence changes at sites that have homology to the sgRNA cognates is thus important to enable clinical translation of the gene editing technology. Possible off-target sites were thus identified for SaCas9-sgRNA-8 using the Off-spotter online tool^18^. Ten sites were predicted as being the most likely sequences where unintended action of SaCas9-sgRNA-8 would occur. The top three sites were verified by additional predictive tools, namely COSMID^27^ and Cas-Offinder tools^28^ (Supp. Fig. 3). After transfection of HEK293T cells with plasmids encoding SaCas9 and the sgRNA-8 cassette, the T7E1 assay failed to detect indels at the identified off-target sites (Fig. 5). To expand the analysis of non-specific action of the anti-HBV RGN, we also took into account the importance of the seed sequence^29^. This element comprises nucleotides at the 3’ end of the sgRNA and is located adjacent to the protospacer adjacent motif (PAM). Previous work using an objective off-target assay that captured all possible break sites within the genome showed that the majority of sites maintained complementarity with the first 3 bp of the seed^16^. An additional ten most likely off-target sites, which maintained the 3 bp seed, were thus identified using the Off-Spotter tool (Supp. Fig. 3). As before, no indels were observed at these sites using the T7E1 nuclease assay (Fig. 5a). Next generation sequencing (NGS) was carried out to achieve more sensitive detection of mutagenesis by sgRNA-8. Sites 1, 2 and 3 (OT1, OT2 and OT3), which were predicted by the three bioinformatics tools to be the most likely off-target sites, were subjected to the analysis (Fig. 5b). Evaluation of sequences extracted from HepG2.2.15 cells was performed on day 21 after transduction with AAVs. Data showed presence of indels at approximately 23% of HBV target sites, which is similar to the quantification using ddPCR^TM^ (Fig. 3b). Conversely NGS showed mutagenesis at less than 0.05% of the off-target sites (Fig. 5b). Low level mutagenesis of HBV DNA extracted from HBV-infected hNTCP cells corroborated analysis using the T7E1 and ddPCR^TM^ assays (Fig. 4g and h). Limited detectable sequence changes at the most likely predicted off-target sites of sgRNA-8 suggest that the guide functions specifically when used in conjunction with SaCas9.

## Discussion

Chronic infection with HBV remains a significant global health problem. Although current treatments provide some benefits to carriers of the virus, licensed drugs rarely eliminate the stable viral replication intermediate comprising cccDNA. Gene-editing technologies potentially provide the means to inactivate cccDNA permanently. However, current efforts to develop gene-editing technologies for HBV treatment are hampered by difficulties with adequate delivery efficiency. ssAAVs are popular vectors that show promise for many applications of gene therapy, but a drawback of these vectors is the limited size of the transgenes that they may accommodate. DNA sequences encoding dimeric ZFNs^6^, TALENs^7^ and Sp RGN components exceed the limit of approximately 4.5 kb. A combination of ssAAVs, which is not ideal, is thus usually required to deliver the complete gene editors. In this study, we have demonstrated the feasibility of using well-characterized ssAAVs for delivery of two cassettes comprising a complete HBV-targeting SaCas9 and guide assembly.

In an unusually compact arrangement of the HBV genome, protein-coding sequences overlap with each other and encompass the entire 3.2 kb that make up the viral genome (Fig. 1a). Use of different reading frames enables viral DNA sequences to encode more than one protein. Moreover, *cis* elements that are responsible for regulating gene expression are embedded within the protein coding sequences, adding to the multifunctional nature of the viral genome. This highly economical use of its DNA constrains sequence plasticity of HBV. The sequence of HBV that was targeted in this study spanned the *S* ORF, which overlaps *P* in its entirety. Mutations introduced through action of gene editors will therefore change both Surface and Polymerase proteins to effect highly efficient inhibition of viral replication. Analysis using T7E1 and drop-off ddPCR^TM^ assays confirmed mutation of the viral DNA with concomitant decrease in HBV replication in HepG2.2.15 cells. Interestingly the finding in hNTCP-HepG2 cells was different. Rather than targeted mutation, a marked decrease in the concentration of cccDNA was demonstrated. This bias towards eradication, and low indel detection, of episomal DNA has been noted by others when using SpCas9 to target HBV^30^. The effect has been attributed to delayed repair of cleaved circular DNA, thereby enhancing its degradation^10^. Although indels have been demonstrated in cccDNA, the effect is thought to occur when CRISPR/Cas9-treated cells contain high concentrations of cccDNA^11^. The fate of mutated cccDNA within a cell is not established. If the disabled cccDNA is eliminated, the risk of genotoxic integration into the host genome would be diminished.

Specificity of gene editors is crucial to their safety and clinical utility. Evaluating off-target action and deciding on acceptable, if any, unintended action is difficult. Use of sensitive unbiased methodology is essential and assays such as BLESS^16^ and Guide-seq.^31^ may be capable of identifying global genomic off-target effects with sufficient depth to inform decisions about safety of particular gene editors. Analyses of SpCas9 have shown that the first generation gene editors may be prone to unintended off-target cleavage (reviewed in ref. 32). This prompted investigation aimed at improving specificity, which amongst other methods entailed truncation of guides^33^ and rational engineering of the SpCas9 endonuclease^34^. The SaCas9 has an improved on-target profile when compared to that of SpCas9^16^. Combination of SaCas9 with its guide may be intrinsically more specific for an intended target than the RGN derived from *S*. *pyogenes*, and reasons for this include the following: 1) sgRNAs derived from *S*. *aureus* are less tolerant of mismatches; 2) an extended PAM sequence needed for SaCas9 binding reduces non-specific interactions; and 3) there is a requirement for a longer seed sequence. Furthermore, modifications have recently been made to SaCas9 to reduce non-specific interactions with DNA, which increases the dependence of cleavage on interaction of the guide with its cognate^34^. These features indicate that the good fidelity of repurposed SaCas9 may make it an RGN with therapeutic potential.

ssAAVs are being developed to treat several hereditary liver diseases, and they show promise in a clinical trial^35^. Accommodating sequences encoding all components of *S*. *aureus* - derived CRISPR*/*Cas9 in a recombinant ssAAVs is very convenient. The good anti-HBV efficacy demonstrated here suggests that these vectors are suitable for evaluation *in vivo* and may also be applicable to permanently eradicating chronic HBV infection

## Materials and Methods

### Plasmids encoding SaCas9 and HBV target sequences

A sgRNA cloning cassette was generated from a gBLOCK (IDT, Coralville, IA, USA) containing a *Bbs*I site for sgRNA insertion (Supp. Table 2). The gBLOCK was amplified using a U6-F and tracrRNA-R primer set (Supp. Table 3) with KAPA Taq ReadyMix (Roche, Basel, Switzerland) and cloned into pTZ57R/T using the insTAclone PCR cloning Kit according to manufacturer’s instructions (Thermo Fisher, MA, USA). DNA encoding targeting sequences of the sgRNAs were synthesized as oligonucleotides and inserted into the *Bbs*I site of pTZ-SaU6-sgRNA. Sequences were verified using standard Sanger sequencing procedures with the U6-F primer (Supp. Table 2).

The pAAV-CMV-SaCas9-Bgh-U6sgRNA expression vector was kindly donated by Dr Feng Zhang^16^. The pAAV-CMV-dSaCas9 vector was generated using Gibson Assembly® (NEB, MA, USA) to insert synthesized DNA gBLOCKs (IDT, IA, USA) harboring the D10A and H557A mutations (Supp. Table 3). Nickase intermediates were used to generate a catalytically dead double-mutant dSaCas9. To insert HBV sgRNA-8 into the ssAAV vector, the pTZ-U6-sgRNA-HBV was digested with *Kpn*I and *Not*I and the purified fragments were ligated into a similarly digested pAAV-CMV-SaCas9-Bgh vector to generate the pAAV-CMV-SaCas9-Bgh-U6-sgRNA-HBV. The HBV replication competent plasmid, pCH-9/3091, has been described previously^19^. HBV-1.3 × A1-a, HBV-1.3 × A2 and HBV-1.3 × D3 were kindly donated by Prof. Anna Kramvis^36^ and HBV-1.3 × A-b was generated using similar standard procedures. Preparation of pCMV-GFP has been previously described^37^.

### Cell culture and transfections

Human embryonic kidney (HEK293T), Huh7 and HepG2.2.15 cell lines were maintained in Dulbecco’s modified Eagle’s medium (DMEM; BioWhittaker, MD, USA) supplemented with 10% fetal calf serum (FCS) (Thermo Fisher, MA, USA). The hNTCP-HepG2 cells^38^ were maintained in DMEM/F12 + GlutaMAX™-I medium (Thermo Fisher, MA, USA) supplemented with 10% FCS, 10 mM HEPES, 0.05 mM Hydrocortisone, 0.005 mg/ml insulin, and 0.4 mg/ml G418 (DMEM/F12-complete medium). Unless otherwise stated, the cell lines were cultured at 37 °C with 5% CO_2_.

### Screening of anti-HBV sgRNAs

For HBV knockdown and off-target analysis, HEK293T or Huh7 cells were seeded at 40,000 cells per well in 48-well plates, 24 hours prior to transfection. The cells were transfected with 200 ng of SaCas9 or dSaCas9 vector with an equal amount of pTZ-U6-sgRNA using Lipofectamine® 3000 according to the manufacturer’s instructions (Invitrogen™, Thermo Fisher, MA, USA). Forty eight hours after transfection HBsAg was measured in the supernatant using the Monolisa™ HBs Ag ULTRA kit (Bio-Rad, CA, USA). DNA was extracted using the KAPA Express Extract Kit and the genomic target sites amplified from 1 μl of lysate using primers described in Supp. Table 4. The KAPA2G Robust HotStart ReadyMix (Roche, Basel, Switzerland) was used for PCR. A T7E1 assay was performed on the amplicons as described below.

### ssAAV production

ssAAVs were produced using an in-house protocol. Briefly, five 15 cm^2^ plates were seeded with 3.75 × 10^6^ HEK293T cells 72 hours prior to transfection. The cells were transfected with 10 μg of Adenohelper plasmid, 6 μg pAAV-CMV-SaCas9-Bgh-U6-sgRNA vector and 8 μg of Rep-Cap2 plasmid (AAV2) with PEI MAX at a 1:5 ratio of DNA to PEI. The Adenohelper and Rep-Cap2 plasmids were kindly provided by Dr. Dirk Grimm. The pAAV-CAG-GFP control vector was obtained from Addgene (#11150). The supernatants were collected on days 1, 3, 5 and 7 after transfection, clarified through 0.22 micron filters and the ssAAVs then precipitated using PEG 8000 after overnight incubation at 4 °C. The precipitated virus was pelleted by centrifugation. In parallel, the AAV-containing cells were lysed using freeze-thaw cycles and sonication in viral lysis solution. After centrifugation to clear the lysate, the supernatant was used to resuspend the PEG-precipitated ssAAV pellet. ssAAVs were separated on an Iodixanol gradient and the fraction containing the ssAAVs (40%) was collected and stored at −80 °C until needed.

### Concentrating infectious HBV particles

HepG2.2.15 cells were seeded at 50% confluence in 175 cm^2^ flasks and the supernatant thereafter collected on days 3 and 5. The supernatant was clarified through a 0.2 micron filter and the HBV particles were PEG-precipitated after overnight incubation at 4 °C. The precipitated virions were pelleted by centrifugation at 4 °C at 4,000 × g for 30 minutes and resuspended in 200 µl of virus concentrating solution. The concentrated virus was quantified by qPCR using ssoFAST Eva Green supermix (Bio-Rad, CA, USA) with primers complementary to the *S* region of HBV (Supp. Table 4). Copies of VPEs were determined using the Acrometrix™ HBV panel to generate a standard curve (Thermo Fisher, MA, USA).

### ssAAV transduction of HBV-producing cell lines

The HepG2.2.15 line was seeded at 40,000 cells per well in a 48-well plate and transduced with 1 × 10^5^ genome copies (GC) of ssAAV per cell. Cells were maintained at 30 °C and 5% CO_2_. The hNTCP-HepG2 cells were seeded in DMEM/F12 complete medium at 20,000 cells per well in a 96-well plate. The medium was replaced after 24 hours with complete media containing 3% dimethyl sulfoxide (DMSO) and incubated for 6 hours. Infection was carried out using 3 × 10^3^ GC of HBV per cell in DMEM/F12 complete medium containing 3% DMSO and 4% PEG-8000 (Sigma, MO, USA). Sixteen hours after HBV infection the cells were washed three times with PBS and medium containing 4 × 10^5^ GC of ssAAV-SaCas9-U6sgRNA-8 per cell added. AAV transductions were repeated on days 3 and 6 with both the HepG2.2.15 and hNTCP-HepG2 cells.

### Quantification of HBV viral particle equivalents

HBV genomes were extracted from 50 μl of culture supernatant using the QIAamp DNA Blood Mini Kit (Qiagen, Hilden, Germany). Five microliters of extracted HBV DNA was analyzed using qPCR with FastStart Universal SYBR qPCR Master (Roche, Basel, Switzerland). Quantification of VPEs was carried out as described above.

### Detection and quantification of RNA

Total RNA was extracted with TRIzol (Thermo Fisher, MA, USA) following procedures recommended by the manufacturer. One microgram of RNA was reverse transcribed using the Quantitect Reverse Transcription Kit (Qiagen, Hilden, Germany), and 1 μl of the cDNA was used in a qPCR with the FastStart Universal SYBR Master (Roche, Basel, Switzerland). Concentrations of SaCas9 and HBV RNA were determined relative to mRNA of *GAPDH*, while the sgRNAs were measured relative to hU6snRNA using primers described in Supp. Table 4.

### Preparation and quantification of HBV cccDNA

Total cellular DNA was extracted using QIAamp DNA Blood Mini Kit (Qiagen, Hilden, Germany) and 200 ng of extracted DNA was subjected to plasmid-safe digestion (Epicenter, WI, USA) in a total reaction volume of 20 μl. The extraction procedure, with DNA from HBV transgenic mice as control for elimination of non-cccDNA, has been described previously^7, 24^. Digestions were carried out for 16 hours at 37 °C and the enzyme was inactivated following incubation at 70 °C for 30 min. Five microliters of treated DNA was amplified using FastStart Universal SYBR Master (Roche, Basel, Switzerland) with *S* primers (Supp. Table 4) and HBV DNA quantified as described earlier.

### Detection of Indels

The target site of the sgRNA was amplified using KAPA2G Robust HotStart ReadyMix (Roche, Basel, Switzerland) with *S* primers (Supp. Table 4) and either 200 ng of extracted DNA or 5 μl of plasmid-safe treated DNA as the template. The PCR products were purified using a GeneJET PCR purification kit (Thermo Fisher, MA, USA), and 400 ng of the purified products were subjected to the T7E1 assay as previously described^7^. For TIDE analysis, the PCR products were analyzed by Sanger sequencing (Inqaba Biotec, Pretoria, South Africa) and the chromatograms analyzed using the TIDE online tool^23^.

### Drop-Off ddPCR^TM^

Drop-off assays were performed using the ddPCR^TM^ instrument from Bio-Rad (Hercules, CA, USA) as described before^39^. Briefly, either 200 ng of total extracted DNA or 5 μl of plasmid-safe treated DNA was mixed with ddPCR™ supermix. Primers specific for the target regions were included together with a FAM-conjugated reference probe and HEX-conjugated probe for the sgRNA-8 cognate (Supp. Tables 4 and 5). The mixture was transferred to a DG8™ cartridge and droplets were generated using 70 μl of ddPCR™ oil for probes. The droplets were transferred to a 96-well plate and the plate sealed. DNA was amplified after initial denaturation at 95 °C for 10 minute, then 40 cycles of 95 °C for 30 seconds, 55 °C for 15 seconds and 72 °C for 1 minute and a final incubation at 98 °C for 10 min. The droplets were analyzed on a QX200™ droplet reader using QuantaSoft™ software. Total intracellular HBV DNA was determined as an absolute count.

### Off-target site analysis

Off-target sites within the host genome were identified using the Off-spotter tool^18^ for a sgRNA-8 recognition sequences with or without a 3 bp seed prediction. DNA was extracted from transiently transfected HEK293T cells and the target sites amplified using Kapa2G Robust HotStart ReadyMix (Roche, Basel, Switzerland) with primers specific for the predicted sites (Supp. Table 4). The T7E1 assays were performed as described earlier.

### Next-generation sequencing

The on-target and selected off-targets were amplified using Q5 hotstart high-fidelity PCR master mix (NEB, MA, USA). Amplicons were purified using the QiaQuick PCR purification kit (Qiagen, Hilden, Germany). Samples were sequenced using an Illumina instrument (HiSeq. 2500) and processing was outsourced to Inqaba Biotec (Pretoria, South Africa). The Fastq files were analyzed using the CRISPResso online indel prediction tool^40^.

### Statistical analysis

Statistical calculations were performed using GraphPad Prism 4 software (GraphPad, CA, USA). Comparisons were determined using an unpaired two-tailed Student’s t-test and differences were considered statistically significant when *p* was less than 0.05.

### Data Availability Statement

Materials, data and associated protocols will be made available on request.

## Electronic supplementary material

Supplementary Information
