# Supplementary material for: ssAAVs containing cassettes encoding SaCas9 and guides targeting hepatitis B virus inactivate replication of the virus in cultured cells
Source: Sci Rep. 2017 Aug 7;7:7401. doi: 10.1038/s41598-017-07642-6 (PMC5547162; doi:10.1038/s41598-017-07642-6)
Supplement: Supplementary file 1 — Supplementary Information [file 41598_2017_7642_MOESM1_ESM.doc]

**Supplementary Data**

**ssAAVs containing cassettes encoding SaCas9 and guides targeting hepatitis B virus inactivate replication of the virus in cultured cells**

Tristan Scott^1,2^, Buhle Moyo^1^, Samantha Nicholson^1^, Mohube Betty Maepa^1^, Koichi Watashi^3^, Abdullah Ely^1^, Marc S. Weinberg^1^ and Patrick Arbuthnot^1*^

^1^ Wits/SAMRC Antiviral Gene Therapy Research Unit, Health Sciences Faculty, University of the Witwatersrand, Johannesburg, South Africa

^2^ HIV Pathogenesis Research Unit, Health Sciences Faculty, University of the Witwatersrand, Johannesburg, South Africa

^3^ National Institute of Infectious Diseases, Department of Virology II, Tokyo Japan

* Corresponding author

Email: [Patrick.Arbuthnot@wits.ac.za](mailto:Patrick.Arbuthnot@wits.ac.za)

Tel +27 11 717 2365

Fax +27 11 717 2395

Wits/SAMRC Antiviral Gene Therapy Research Unit, Health Sciences Faculty, University of the Witwatersrand, Johannesburg, South Africa.

HBsAg (relative)

NS Guide

HIV-sgRNA

HBV-8-sgRNA

0

50

100

150

pCH9/3091

HBV1.3×A1-a

HBV1.3×A1-b

HBV1.3×A2

HBV1.3×D3

**Supplementary Figure 1. HBV-8-sgRNA does not inhibit sub-genotypes of HBV with a catalytically dead *sa*Cas9.** Huh7s cells were transfected with a dsaCas9 vector with sgRNA-8 targeted to greater-than-genome length molecular clones (1.3×) for HBV sub-genotypes A1, A2 and D3. The concentrations of HBV S antigen were measured using an ELISA and data was presented relative to the values for the mean for a non-specific sgRNA sequence. An sgRNA targeted to HIV (HIV-sgRNA) was included as an additional negative control. Data are represented as the means and the error bars indicate standard deviation. HBsAg (relative)


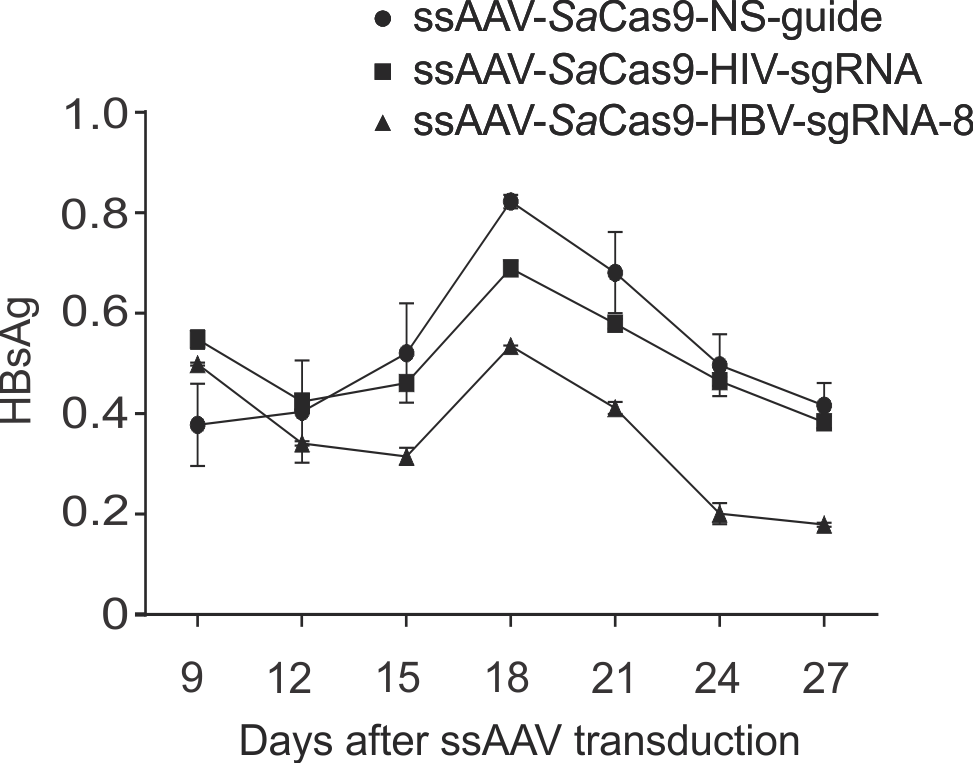


**Supplementary Figure 2. Absolute levels of HBV S antigen following infection of hNTCP-HepG2 cells.** Infection with HBV was carried out 24 hours prior to transduction of hNTCP cells with ssAAVs. Concentrations of HBsAg in supernatants were determined using ELISA. A non-specific sgRNA (NS) or an sgRNA targeted to HIV (HIV-sgRNA) were included as an additional negative control. Data are represented as the means and the error bars indicate standard deviation.

**a**

**b**

**Supplementary Figure 3. Prediction of off-target sites recognized by HBV-sgRNA-8.** Human genomic off-targets sites were determined **(a)** for sgRNA-8 without a “seed” or **(b)** with 3 bp seed. Mismatches are denoted in red. COSMID or Cas-Offinder was used as additional tools for the top three “no seed” predictions. Off-target scores for COSMID are indicated in parentheses.

**Supplementary Table 1. Sequences of multiple HBV sub-genotypes (A-H) align with HBV-sgRNA-8**.

| HBV sub-genotype | Sequence 5'-3' |
| --- | --- |
| HBV 1.3× A1-a | CAAGAATCCTCACAATACCG**CAGAGT** |
| HBV 1.3× A1-b | CAAGAATCCTCACAATACCG**CAGAGT** |
| HBV 1.3× A2 | CAAGAATCCTCACAATACCG**CAGAGT** |
| HBV 1.3× D3 | CAAGAATCCTCACAATACCG**CAGAGT** |
| HBV ayw HepG2.2.15 U95551 | CAAGAATCCTCACAATACCG**CAGAGT** |
| HBV A1 AY233288 | CAAGAATCCTCACAATACCG**CAGAGT** |
| HBV A2 X70185 | CAAGAATCCTCACAATACCG**CAGAGT** |
| HBV A3 AB194950 | CAAGAATCCTCACAATACCG**CAGAGT** |
| HBV B1 AB073851 | CAAAAATCCTCACAATACCA**CAGAGT** |
| HBV B2 AF121245 | CAAAAATCCTCACAATACCA**CAGAGT** |
| HBV B3 EF473977 | CAAAAATCCTCACAATACCA**CAGAGT** |
| HBV B4 AB115551 | CAAAAATCCTCACAATACCA**CAGAGT** |
| HBV B5 AB219428 | CAAAAATCCTCACAATACCA**CAGAGT** |
| HBV B6 AB287316 | CAAAAATCCTCACAATACCG**CAGAGT** |
| HBV C1 DQ089760 | CAAGAATCCTCACAATACCA**CAGAGT** |
| HBV C2 AB365451 | CAAGAATCCTCACAATACCA**CAGAGT** |
| HBV C3 X75665 | CAAAAATCCTCACAATACCA**CAGAGT** |
| HBV C4 AB048704 | CAAGAATCCTCACAATACCT**CAGAGT** |
| HBV C5 EU410079 | CAAAAATCCTCACAATACCA**CAGAGT** |
| HBV C6 AB493837 | CAAGAATCCTCACAATACCA**CAGAGT** |
| HBV D1 AB246348 | CAAGAATCCTCACAATACCG**CAGAGT** |
| HBV D2 EU594416 | CAAGAATCCTCACAATACCG**CAGAGT** |
| HBV D3 EU594435 | CAAGAATCCTCACAATACCG**CAGAGT** |
| HBV D4 AB033559 | CAAGAATCCTCACAATACCG**CAGAGT** |
| HBV E AM494708 | CAAAAATCCTCACAATACCG**CAGAGT** |
| HBV F1 AY090458 | CAAAAATCCTCACAATACCA**CAGAGT** |
| HBV F2 AY311369 | CAAAAATCCTCACAATACCA**CAGAGT** |
| HBV F3 AB036909 | CAAAAATCCTCACAATACCA**CAGAGT** |
| HBV F4 AF223965 | CAAAAATCCTCACAATACCA**CAGAGT** |
| HBV G AB375168 | CAAGAATCCTCACAATACCG**CAGAGT** |
| HBV H AY090457 | CAAAAATCCTCACAATACCA**CAGAGT** |
| pCH-9/3091 | CAAGAATCCTCACAATACCG**CAGAGT** |
| **saCas9 guide** |  |
| **HBV-8-sgRNA** | CAAGAAUCCUCACAAUACCG |

Mismatches are indicated in red and the PAM sequence (5’ NNGRRT 3’) is in bold.

**Supplementary Table 2. Primers and gBLOCKs used for the generation of the U6-sgRNA-BbsI cloning vector and dead saCas9.**

| gBlock/primer | Sequence 5’-3’ * | Length (nt) |
| --- | --- | --- |
| U6-F | **GGTACC**TGAGGGCCTATTTCCCATGATTC | 29 |
| TracrRNA-R | **GCGGCCG**CAAAAATCTCGCCAA | 22 |
| U6-sgRNA-unmodified tracrRNA | **GGTACC**GAGGGCCTATTTCCCATGATTCCTTCATATTTGCATATACGATACAAGGCTGTTAGAGAGATAATTGGAATTAATTTGACTGTAAACACAAAGATATTAGTACAAAATACGTGACGTAGAAAGTAATAATTTCTTGGGTAGTTTGCAGTTTTAAAATTATGTTTTAAAATGGACTATCATATGCTTACCGTAACTTGAAAGTATTTCGATTTCTTGGCTTTATATATCTTGTGGAAAGGACGAAACACCGGGTCTTCGAGAAGACCTGTTTAAGTACTCTGTGCTGGAAACAGCACAGAATCTACTTAAACAAGGCAAAATGCCGTGTTTATCTCGTCAACTTGTTGGCGAGATTTTT**GCGGCCGC** | 391 |
| RuvCI mutation (D10A) gBLOCK | CAGAGCTCTCTGGCTAACTACCGGTGCCACCATGGCCCCAAAGAAGAAGCGGAAGGTCGGTATCCACGGAGTCCCAGCAGCCAAGCGGAACTACATCCTGGGCCTGGCCATCGGCATCACCAGCGTGGGCTACGGCATCATCGACTACGAGACACGGGACGTGATCGATGCCGGCGTGCGGCTGTTCAAAGAGGCCAACGTGGAAAACAACGAGGGCAGGCGGAGCAAGAGAGGCGCCAGAAGGCTGAAGCGGCGGAGGCGGCATAGAATCCAGAGAGTGAAGAAGCTGCTGTTCGACTACAACCTGCTGACCGACCACAGCGAGCTGAGCGGCATCAACCCCTACGAGGCCAGAGTGAAGGGCCTGAGCCAGAAGCTGAGCGAGGAAGAGTTCTCTGCCGCCCTGCTGCACCTGGCCAAGAGAAGAGGCGTGCACAACGTGAACGAGGTGGAAGAGGACACCGGCAACGAGCTGTCCACCAGAGAGCAGATCAGCCGGAACAGCAAGGCCCTGGAAGAGAAATACGTGGCCGAACTGCAGCTGGAACGGCTGAAGAAAGACGGCGAAGTGCGGGGCAGCATCAACAGATTCAAGACCAGCGACTACGTGAAAGAAGCCAAACAGCTGCTGAAGGTGCAGAAGGCCTACCACCAGCTGGACCAGAGCTTCATCGACACCTACATCGACCTGCTGGAAACCCGGCGGACCTACTATGAGGGACCTGGCGAGGGCAGCCCCTTCGGCTGGAAGGACATCAAAGAATGGTACGAGATGCTGATGGGCCACTGCACCTACTTCCCCGAGGAACTGCGGAGCGTGAAGTACGCCTACAACGCCGACCTGTACAACGCCCTGAACGACCTGAACAATCTCGTGATCACCAGGGACGAGAACGAGAAGCTGGAATATTACGAGAAGTTCCAGATCATCGAGAACGTGTTCAAGCAGAAGAAGAAGCCCACCCTGAAGCAGATCGCCAAAGAAATCCTCGTGAACGAAGAGGATATTAAGGGCTACAGAGTGACCAGCACCGGCAAGCCCGAGTTCACCAACCTGAAGGTGTACCACGACATCAAGGACATTACCGCCCGGAAAGAGATTATTGAGAACGCCGAGCTGCTGGATCAGATTGCCAAGATCCTGACCATCTACCAGAGCAGCGAGGACATCCAGGAAGAACTGACCAATCTGAACTCCGAGCTGACCCAGGAAGAGATCGAGCAGATCTCTAATCTGAAGGGCTATACCGGCACCCACAACCTGAGCCTGAAGGCCATCAACCTGATCCTGGACGAGCTGTGGCACACCAACGACAACCAGATCGCTATCTTCAACCGGCTGAAGCTGGTGCCCAAGAAGGTGGACCTGTCCCAGCAGAAAGAGATCCCCACCACCCTGGTGGACGACTTCATCCTGAGCCCCGTCGTGAAGAGAAGCTTCATCCAGAGCATCAA | 1455 |
| HNH mutation (H557A) gBlock | CAGAAAGAGATCCCCACCACCCTGGTGGACGACTTCATCCTGAGCCCCGTCGTGAAGAGAAGCTTCATCCAGAGCATCAAAGTGATCAACGCCATCATCAAGAAGTACGGCCTGCCCAACGACATCATTATCGAGCTGGCCCGCGAGAAGAACTCCAAGGACGCCCAGAAAATGATCAACGAGATGCAGAAGCGGAACCGGCAGACCAACGAGCGGATCGAGGAAATCATCCGGACCACCGGCAAAGAGAACGCCAAGTACCTGATCGAGAAGATCAAGCTGCACGACATGCAGGAAGGCAAGTGCCTGTACAGCCTGGAAGCCATCCCTCTGGAAGATCTGCTGAACAACCCCTTCAACTATGAGGTGGACGCCATCATCCCCAGAAGCGTGTCCTTCGACAACAGCTTCAACAACAAGGTGCTCGTGAAGCAGGAAGAAAACAGCAAGAAGGGCAACCGGACCCCATTCCAGTACCTGAGCAGCAGCGACAGCAAGATCAGCTACGAAACCTTCAAGAAGCACATCCTGAATCTGGCCAAGGGCAAGGGCAGAATCAGCAAGACCAAGAAAGAGTATCTGCTGGAAGAACGGGACATCAACAGGTTCTCCGTGCAGAAAGACTTCATCAACCGGAACCTGGTGGATACCAGATACGCCACCAGAGGCCTGATGAACCTGCTGCGGAGCTACTTCAGAGTGAACAACCTGGACGTGAAAGTGAAGTCCATCAATGGCGGCTTCACCAGCTTTCTGCGGCGGAAGTGGAAGTTTAAGAAAGAGCGGAACAAGGGGTACAAGCACCACGCCGAGGACGCCCTGATCATTGCCAACGCCGATTTCATCTTCAAAGAGTGGAAGAAACTGGACAAGGCCAAAAAAGTGATGGAAAACCAGATGTT | 902 |

* Bold sequences represent KpnI and NotI restriction sites for cloning of the sgRNAs into the pAAV-saCas9 vector. Underlined sequences represent mutated sequence to generate defective RuvCI and HNH sites.

**Supplementary Table 3. Oligomers used for generation of the anti-HBV sgRNAs.**

| Oligomer | Sequence 5’-3’ * | Length (nt) |
| --- | --- | --- |
| HBV-1-sgRNA (+) | CACCGTCTAGACTCTGCGGTATTGT | 25 |
| HBV-1-sgRNA (-) | AAACACAATACCGCAGAGTCTAGAC | 25 |
| HBV-2-sgRNA (+) | CACCGCCAACTTGTCCTGGTTATCG | 25 |
| HBV-2-sgRNA (-) | AAACCGATAACCAGGACAAGTTGGC | 25 |
| HBV-3-sgRNA (+) | CACCGAAGAAGATGAGGCATAGCAG | 25 |
| HBV-3-sgRNA (-) | AAACCTGCTATGCCTCATCTTCTTC | 25 |
| HBV-4-sgRNA (+) | CACCGCCCGTTTGTCCTCTAATTC | 24 |
| HBV-4-sgRNA (-) | AAACGAATTAGAGGACAAACGGGC | 24 |
| HBV-5-sgRNA (+) | CACCGCGGGGTTTTTCTTGTTGAC | 24 |
| HBV-5-sgRNA (-) | AAACGTCAACAAGAAAAACCCCGC | 24 |
| HBV-6-sgRNA (+) | CACCGACCCCTTCTCGTGTTACAGG | 25 |
| HBV-6-sgRNA (-) | AAACCCTGTAACACGAGAAGGGGTC | 24 |
| HBV-7-sgRNA (+) | CACCGTGATTGGAGGTTGGGGACT | 24 |
| HBV-7-sgRNA (-) | AAACAGTCCCCAACCTCCAATCAC | 24 |
| HBV-8-sgRNA (+) | CACCGCAAGAATCCTCACAATACCG | 25 |
| HBV-8-sgRNA (-) | AAACCGGTATTGTGAGGATTCTTGC | 25 |
| HBV-9-sgRNA (+) | CACCGAAAATTGAGAGAAGTCCACC | 25 |
| HBV-9-sgRNA (-) | AAACGGTGGACTTCTCTCAATTTTC | 25 |
| HBV-10-sgRNA (+) | CACCGAGTTGGAGGACAAGAGGTTG | 25 |
| HBV-10-sgRNA (-) | AAACCAACCTCTTGTCCTCCAACTC | 25 |
| HIV-sgRNA (+) | CACCGTTCTACAAGGGACTTTCCGC | 25 |
| HIV-sgRNA (-) | AAACGCGGAAAGTCCCTTGTAGAAC | 25 |

* Underlined sequences represent overhangs for cloning into a BbsI digested U6-gRNA vector.

**Supplementary Table 4. Primers and probes used for qRT-PCR, AAV quantification, drop-off and T7E1 assays.**

| Primer / Probe | Sequence 5’-3’ | Length (nt) |
| --- | --- | --- |
| HBV S F | TGCACCTGTATTCCCATC | 18 |
| HBV S R | CTGAAAGCCAAACAGTGG | 18 |
| HBV pre-core/genomic F | ACCACCAAATGCCCCTAT | 18 |
| HBV pre-core/genomic R | TTCTGCGACGCGGCGA | 16 |
| Control gRNA F | GGGTCTTCGAGAAGACCTGT | 20 |
| HBV-8 gRNA F | GCAAGAATCCTCACAATACCG | 21 |
| tracrRNA F | GTTTAAGTACTCTGGAAACAGAATCT | 26 |
| tracrRNA R | AACAAGTTGACGAGATAAAC | 20 |
| saCas9 F* | CCGCCCGGAAAGAGATTATT | 20 |
| saCas9 R* | CGGAGTTCAGATTGGTCAGTT | 21 |
| AAV saCas9 probe | FAM-AGCTGCTGGATCAGATTGCCAAGA-BHQ1 | 24 |
| GAPDH F | GAAGGTGAAGGTCGGAGTC | 19 |
| GAPDH R | GAAGATGGTGATGGGATTTC | 20 |
| GAPDH Plasmid-safe R | AACTACCCATGACTCAGCTTCTCC | 24 |
| hU6-snRNA F | CTCGCTTCGGCAGCACA | 17 |
| hU6-snRNA R | AACGCTTCACGAATTTGCGT | 20 |
| gRNA-8 T7E1 F | CATGCAGTGGAATTCCACAACCTT | 24 |
| gRNA-8 T7E1 R | CTGGAATTAGAGGACAAACGGGCA | 24 |
| ddPCR F | CCTTCTCGTGTTACAGGCG | 19 |
| ddPCR R | CCAAGACACACGGTAGTTCC | 20 |
| gRNA-8 probe | HEX-CCTCACAATACCGCAGAGTCTA-BHQ1 | 22 |
| HBV Reference probe | FAM-TGAGAGAAGTCCACCACGA-BHQ1 | 19 |
| AAV GFP F | GACGACGGCAACTACAAGA | 19 |
| AAV GFP R | GATGCCCTTCAGCTCGAT | 18 |
| GFP probe | FAM-AGGTGAAGTTCGAGGGCGACAC-BHQ1 | 22 |

* Primers used for both detection of *saCas9* mRNA and quantification of expression cassette-containing AAVs.

**Supplementary Table 5. Primer sets used to amplify genomic off-target sites**

| Primer | Sequence 5’-3’ | Length (nt) |
| --- | --- | --- |
| OT-1 No seed F | AGGAAGAGCAATGGACTAGGAGG | 25 |
| OT-1 No seed R | CCCGATGATCTGCAGAATATAACCAC | 23 |
| OT-2 No seed F | CAGGTACCTCAGTTGGAAATGCAG | 25 |
| OT-2 No seed R | TCTCTAGTTGACAGCACTTTTGCTC | 25 |
| OT-3 No seed F | AGGAAAGATCTATGTTCTAATGCCCAAC | 30 |
| OT-3 No seed R | GCCATGGACCTACATAAGCAGTGG | 25 |
| OT-4 No seed F | GATGTGCAGCTCTTCTAGCACCA | 30 |
| OT-4 No seed R | TTCAGTTGTCTTTGTTTGCAGACATG | 23 |
| OT-5 No seed F | TTTGCAATGAGCCGAGATCGTG | 20 |
| OT-5 No seed R | CTTGGACAGCAACCTCCTCACC | 19 |
| OT-6 No seed F | GTCAGATTGGGCATGGTTCTACAC | 21 |
| OT-6 No seed R | CTACAAATCGCAGCAATAGAGCACT | 22 |
| OT-7 No seed F | CCAGGTGGAATTAAAGGGTAATGTGG | 20 |
| OT-7 No seed R | CCCACCGTACTTTAGAAAACTGGAG | 22 |
| OT-8 No seed F | GCATGAACCACATGCCAATTACAGA | 22 |
| OT-8 No seed R | GGATTAAAGCCATAAGCCACCACAC | 20 |
| OT-9 No seed F | CTCAGTTGGAAATGCAGAAAGCACC | 22 |
| OT-9 No seed R | ATCTCTAGTTGACAGCACTTTTGCTC | 20 |
| OT-10 No seed F | GGTTATGAGTCACTGGATGGATCATGG | 20 |
| OT-10 No seed R | CCATCACCTAAGTATTAAGCTCAGCATGC | 19 |
| OT-1 3bp-seed F | TGACAAGGTAGGAGCTCCTCTTAGC | 23 |
| OT-1 3bp-seed R | CAAGCCACAGGCTTTGTTATGTG | 26 |
| OT-2 3bp-seed F | ATCTTAGTTGCTTCCAAGTTCTGGC | 24 |
| OT-2 3bp-seed R | CTCAACAGCAAGAAAACAAGCAGGC | 25 |
| OT-3 3bp-seed F | ACAATACCTTGTAGAAATATCTCCAAGGTG | 28 |
| OT-3 3bp-seed R | CCTGGGAGATCAGGAGATTAAGAGG | 24 |
| OT-4 3bp-seed F | TTGTTATCAGCCTAAAATTCCACATAGGTG | 23 |
| OT-4 3bp-seed R | AAGCCAGTTCCACCTACACAAGC | 26 |
| OT-5 3bp-seed F | AAGGCAGTTCTGAAAGCTTC | 22 |
| OT-5 3bp-seed R | GGAAGCTTCCTGAACAACC | 22 |
| OT-6 3bp-seed F | CGGTGAAATTGGCCAGTAGAG | 24 |
| OT-6 3bp-seed R | GTAGGTGACAAGAATCTACCAG | 25 |
| OT-7 3bp-seed F | GGCCTTTCAACATACGCAGG | 26 |
| OT-7 3bp-seed R | GTTCCTGTTCACATGGTCTTCC | 25 |
| OT-8 3bp-seed F | GGAAGTGAAGAACAGCGTTTCT | 25 |
| OT-8 3bp-seed R | GGTTGGATGAGCTGTGAAGG | 25 |
| OT-9 3bp-seed F | CCAAAGCTTGATGACATTCTGT | 25 |
| OT-9 3bp-seed R | TTGGTGAGTGGTTGACCAAG | 26 |
| OT-10 3bp-seed F | CTCACCACATTCTCAGTGCC | 21 |
| OT-10 3bp-seed R | GGCCACTCCTGCCTCTACC | 22 |
